# Sustained Effects of Memory and Lifestyle Interventions on Memory Functioning of Older Adults: An 18-Month Follow-Up Study

**DOI:** 10.3389/fnagi.2018.00240

**Published:** 2018-08-07

**Authors:** Agnes S. Chan, Winnie K. Cheung, Michael K. Yeung, Tsz Lok Lee

**Affiliations:** ^1^Department of Psychology, The Chinese University of Hong Kong, Hong Kong, Hong Kong; ^2^Chanwuyi Research Center for Neuropsychological Well-Being, The Chinese University of Hong Kong, Hong Kong, Hong Kong

**Keywords:** older adult, memory complaints, memory intervention, lifestyle intervention, subjective well-being

## Abstract

**Background:** There has been much research devoted to examining the short-term effects of different interventions for improving memory functioning of older adults with memory complaints. Nevertheless, very few studies have examined the long-term effects of these interventions. Thus, the present study compared the sustained effects of a conventional memory intervention (MI) and a Chinese lifestyle intervention on improving memory functioning in older adults.

**Methods:** Twenty-nine older adults who were aged 60 years and older and had memory complaints were recruited. Each completed 10 weekly sessions of the *Dejian* Mind-body Intervention (DMBI; *n* = 11) or MI (*n* = 18) approximately 18 months ago. Participants’ verbal and visual memory functioning and their subjective impression of the changes of their memory performance and physical and psychological health status were evaluated.

**Results:** Results showed significant improvements in memory in both intervention groups at the follow-up assessments when compared with baseline. In addition, older adults in both intervention groups perceived improved memory performance and physical and psychological wellness at follow-up, with the DMBI group reporting significantly greater improvements in physical health compared to the MI group.

**Conclusion:** Altogether, the present study provides supportive evidence that the DMBI and MI might be two effective remedies for older adults to improve or preserve their memory functioning with relatively sustained effects.

## Introduction

Memory complaints that occur in older adults have been shown to be a risk factor for the development of dementia (Jonker et al., 2000; Mitchell et al., 2014). In addition, they have been shown to be associated with reduced physical and mental health well-being (Comijs et al., 2002; Ito et al., 2013; Steinberg et al., 2013). Thus, effective interventions that improve or maintain memory functioning of older adults are clinically valuable. Common interventions for older adults with memory complaints include memory/cognitive training (Gross et al., 2012). An alternative to these approaches is lifestyle intervention (Chan et al., 2014; Yu et al., 2014; Ngandu et al., 2015). While memory training enhances memory functioning of older adults by teaching internal and external mnemonic strategies to facilitate encoding and retrieval of information (Gross et al., 2012), lifestyle interventions involve adoption of exercise, diet and at least one other component (e.g., counseling, smoking cessation, behavior modification) that aim to reverse pathology and/or delay disease progression by modifying high-risk health habits (American College of Preventive Medicine, 2009).

Recently, we examined the effectiveness of both memory training and lifestyle intervention and found that their immediate effects on improving memory functioning of older adults were comparable. That is, older adults who received either conventional memory intervention (MI) or a Chinese Chan-based lifestyle intervention, namely the *Dejian* Mind-body Intervention (DMBI), showed significant improvements in memory functioning as compared with their baseline performance (Chan et al., 2017). In addition, the effects of the DMBI on improving subjective memory functioning and physical and psychological wellness were demonstrated (Yu et al., 2014; Chan et al., 2017).

Prior independent research suggested significant immediate effects of both memory/cognitive training (Belleville et al., 2006; Calero and Navarro, 2007; Jean et al., 2010; Metternich et al., 2010; Gross et al., 2012; Vermeij et al., 2016) and lifestyle intervention (Chan et al., 2014; Yu et al., 2014; Ngandu et al., 2015) on improving memory functioning of older adults. Some studies reported the effect of MI on memory functioning of older adults with memory complaints can last up to 6 months (Scogin and Prohaska, 1992; Metternich et al., 2008), while the sustained effects of lifestyle intervention remain unknown. Thus, the present study aimed to evaluate the sustained effects (i.e., 18 months) of memory training (i.e., MI) and lifestyle intervention (i.e., DMBI) on older adults with memory complaints. Sustained effects were operationalized as differences in memory functioning measured between follow-up and baseline assessments. In addition, based on the reported relationships between memory complaints and physical and mental health well-being (Comijs et al., 2002; Ito et al., 2013; Steinberg et al., 2013), we also evaluated subjective changes in physical and mental health status.

## Materials and Methods

### Participants

The present research extends a published study that examined the immediate effects of 10 weekly sessions of the DMBI or MI on memory functioning and subjective changes in memory performance and physical and psychological health on community-dwelling older adults aged 60 years and older (DMBI: *n* = 22; MI: *n* = 26; Chan et al., 2017). All the participants were re-contacted approximately 18 months after the intervention sessions. Eleven older adults from the DMBI group and 18 from the MI group returned for follow-up (see Table 1); we failed to contact the others. A chi-squared test showed no significant group difference in the number of participants who returned for follow-up, *χ*^2^ = 1.8, *p* = 0.18. In addition, these follow-up samples appeared to be representative of the original samples. There were no significant differences in demographic characteristics, memory functioning at baseline, or the extent of improvements in objective and subjective memory functioning. There were also no significant differences in subjective physical and psychological health at post-assessment between the older adults who returned for follow-up and those who did not, *p*s ≥ 0.05 (except for the MI group, the older adults who returned for follow-up had significantly greater subjective improvement in overall psychological health than those who did not return, *p* = 0.01).

**Table 1:** Demographic characteristics of the Memory Intervention (MI) and *Dejian* Mind-Body Intervention (DMBI) groups at baseline.

	Group					
	MI (*n* = 18)		DMBI (*n* = 11)			
Variables	*M*	*SD*	*M*	*SD*	*t*/χ^2^	*p*
Age (years)	68.6	6.8	69.1	5.2	0.21	0.84
Sex (male/female)^∧^	4/14		3/8		0.00	1.00
Handedness (right/left)^∧^	17/1		11/0		0.00	1.00
Education (years)	8.7	4.7	9.7	4.3	0.58	0.57
CDRS (raw total score)	131.7	6.9	130.6	7.8	0.40	0.69
BAI	3.6	3.3	3.6	2.9	0.01	0.99
CGDS-SF	1.7	1.6	1.9	1.8	0.38	0.71

*Note. CDRS, the Chinese version of the Mattis Dementia Rating Scale; BAI, Beck Anxiety Inventory; CGDS-SF, the short form of the Chinese Geriatric Depression Scale. ^∧^Chi-squared test with Yates’s continuity correction was performed for distributions violating the sample size assumptions of the chi-squared test*.

### Procedure

All participants underwent a memory assessment identical to that at post-assessment (see Chan et al., 2017, for details): participants were individually assessed on the Hong Kong List Learning Test (HKLLT; Chan, 2006) and the Visual Reproduction subtest of the Wechsler Memory Scale-III (WMS-III-VR; Wechsler, 2005). In addition, they were asked to complete a questionnaire to evaluate perceived changes in their current physical and psychological health and memory performance due to the interventions. All participants provided written informed consent before the study, and it was conducted in accordance with the Helsinki Declaration of the World Medical Association Assembly and approved by the Joint Chinese University of Hong Kong—New Territories East Cluster Clinical Research Ethics Committee.

### Intervention

Detailed descriptions of the DMBI and MI can be found in Chan et al. (2017). In summary, the structure and format of the two intervention groups were designed to parallel each other with respect to duration (i.e., 1.5 h/session), frequency of sessions (i.e., once a week), group size, teaching and learning elements, in-session sharing and discussions. For the DMBI, it was developed based on *Chanwuyi* (i.e., the Chinese *Chan* tradition, martial arts and the *Chan* medical principle) and consisted of three components: (1) to alleviate psychological distress by understanding the root of problems in accordance with the Buddhist philosophy; (2) to enhance physical health by modifying one’s diet to reduce the intake of food that generates excessive internal heat; and (3) to facilitate *Qi* and blood circulation by practicing *Nei Gong* (i.e., mind-body exercises; Chan et al., 2009, 2011a,b, 2012a,b, 2013, 2014; Yu et al., 2014). For the MI, the class structure and content were derived from Troyer et al. (2008) and incorporated three components: (1) to provide psychoeducation on normal and pathological aging and on different types of memory; (2) to teach mnemonic strategies that are well-established in the literature; and (3) to provide at-home assignments to facilitate the daily application of memory strategies.

### Materials and Procedure

The HKLLT (Chan, 2006) and WMS-III-VR (Wechsler, 2005) were administered to evaluate verbal and visual memory functioning, respectively. Both tests require learning certain stimuli followed by a free recall and recognition of these stimuli after time delays. Specifically, the HKLLT required participants to learn a fixed list of 16 Chinese words, which were randomly presented in four semantic categories, through three learning trials. After 10 min and 30 min of delay, participants were asked to recall as many words as possible. A recognition task was presented immediately after the 30-min delay trial. It consisted of a list of 32 items and required participants to judge whether the items were present in the original word list in a yes/no format. In addition, the WMS-III-VR required participants to first learn five sets of geometric forms, shown one at a time for 10 s each, and then to draw them from memory immediately and after 30 min of delay. A recognition trial was presented after the delayed-recall trial. It consisted of 48 items and required participants to judge whether the item was one they were asked to remember earlier in a yes/no format. The WMS-III-VR recall and recognition scores have a test-retest reliability of 0.70–0.73, indicating adequate consistency (Wechsler, 2005). Both the HKLLT (Au et al., 2003) and WMS-III-VR (Salmon et al., 2002) have been shown to be efficient in targeting memory deficits associated with Alzheimer’s disease.

The Chinese version of the Mattis Dementia Rating Scale (CDRS; Chan et al., 2003) was administered to estimate the global cognitive functioning level, and Chinese versions of the Geriatric Depression Scale—Short Form (CGDS; Lee et al., 1993; Liu et al., 1998) and Beck Anxiety Inventory (BAI; Beck et al., 1988) were used to measure participants’ recent levels of depressive and anxiety symptoms, respectively. In addition, participants completed a questionnaire at the post- and follow-up assessments to assess their impressions of the effect of the intervention on their current memory performance as well as physical and psychological status. The questionnaire comprised seven questions, including Overall Physical Health, Mood, Respiratory Function, Gastrointestinal Function, Muscle Flexibility, Overall Psychological Health, Sleep Quality and Memory Performance. They were rated on a 7-point Likert scale ranging from −3 (worsened) to +3 (improved), with higher scores indicating higher levels of subjective improvement. These items were designed based on our previous findings of improved physical fitness, gastrointestinal health, sleep quality and self-rated health following the DMBI in older adults (Yu et al., 2014).

At follow-up, we also asked participants whether they continued to apply intervention methods in daily life. For the DMBI group, we asked participants whether they continued to develop an understanding of the root of problems in accordance with the Buddhist philosophy, to practice Nei Gong and to modify their diets to reduce taking in food that generates excess internal heat (i.e., three methods). For the MI group, we asked participants whether they continued to apply internal mnemonic techniques, such as categorization, spaced retrieval, association, and visual imagery and external memory aids, such as reminder notes (i.e., five methods).

### Data Analysis

For demographic and clinical measures, the DMBI and MI groups were compared using chi-squared tests for categorical variables. Yates’s continuity correction was applied if more than 20% of the cells had expected counts less than five. The groups were compared using independent sample *t*-tests for non-categorical variables. To compare the pattern of changes in memory performance between groups, mixed ANOVA with time (pre-, post-, follow-up) as the within-subject factor and group (DMBI, MI) as the between-subject factor were performed for each performance index. In addition, we performed planned analyses by conducting repeated measures ANOVA with time (pre-, post-, follow-up) as the within-subject factor for each memory functioning index to compare changes in memory performance before intervention (baseline), immediately after the intervention (post) and at 18 months after intervention (follow-up) within each group. Paired *t*-tests (two-sided) were performed if the main effect of time was significant.

Because of small sample size, we performed additional analyses to find out the sample size required to achieve sufficient statistical power for detecting group differences. If the required sample size for a variable is within the range expected for practical significance (e.g., *n* < 91 per group, estimated based on a power of 0.80, an alpha level of 0.05 and the minimum effect size representing a practically significant effect, i.e., $\eta_{\text{p}}^{2}$ = 0.04; Ferguson, 2009), the lack of significant group difference in this variable could be due to an insufficient statistical power, which warrants further studies. Furthermore, to examine whether there were changes in perceived memory, physical health, and psychological status, *t*-tests (two-sided) were performed. All statistical analyses were performed using SPSS 24.0 software (IBM Corporation, Armonk, NY, USA). The significance level was set at 0.05.

## Results

### Changes in Memory Functioning

First, the group differences in changes of memory functioning over time were examined. The results of Group × Time mixed ANOVA showed no significant main effects of group nor Time × Group interactions for any of the HKLLT and WMS-III-VR indices, *F*s ≤ 2.3, *p*s ≥ 0.14. These results suggest that the extent of improvement in memory functioning in the DMBI group was statistically comparable to that of the MI group.

We then performed planned analyses to compare verbal and visual memory functioning at baseline, post-, and follow-up assessments were first compared within each group. For the DMBI group, the results of repeated measures ANOVA showed that the main effects of time were significant for all HKLLT indices, including total learning, 10-min and 30-min delayed recall, and recognition, *F*s > 11.0, *p*s ≤ 0.001, $\eta_{\text{p}}^{2}$ ≥ 0.52 (see Table 2). Paired *t*-tests showed that all indices improved from baseline to post- and follow-up assessments, *p*s ≤ 0.003, *d*s ≥ 1.2. There were no significant differences between post- and follow-up assessments, *p*s > 0.05. In addition, there was a significant main effect of time for the WMS-III-VR delayed recall, *F*_(2,20)_ = 4.0, *p* = 0.04, $\eta_{\text{p}}^{2}$ = 0.29. Paired *t*-tests showed that it significantly improved from baseline to follow-up, *p* = 0.04, *d* = 0.74, suggesting that a significant improvement in visual delayed recall was achieved at follow-up. No other pairwise comparisons were significant, *p*s > 0.05.

**Table 2:** Raw scores of the memory functioning indices before intervention (baseline), immediately (post) and 18 months (follow-up) after intervention in the MI and DMBI groups.

	Group									
	MI (*n* = 18)					DMBI (*n* = 11)				
	Baseline	Post	Follow-up	*F*	*Post hoc t*-contrast	Baseline	Post	Follow-up	*F*	*Post hoc t*-contrast
**HKLLT**										
Total learning	22.5 (5.6)	27.9 (6.9)	29.7 (7.2)	16.9***	Follow-up, Post > Baseline	22.6 (6.2)	28.2 (6.9)	27.8 (7.1)	15.3***	Follow-up, Post > Baseline
10-min delayed recall	7.7 (3.2)	9.9 (3.2)	10.4 (4.1)	13.4***	Follow-up, Post > Baseline	7.2 (3.2)	9.5 (3.3)	9.7 (3.0)	15.2**	Follow-up, Post > Baseline
30-min delayed recall	7.33 (3.6)	9.7 (3.1)	10.5 (3.9)	17.5***	Follow-up, Post > Baseline	6.6 (2.8)	9.8 (3.5)	9.7 (3.1)	13.1***	Follow-up, Post > Baseline
Recognition (discrimination score)	82.3 (15.2)	90.6 (10.6)	89.6 (13.0)	7.0**	Follow-up, Post > Baseline	73.9 (17.6)	87.5 (13.7)	85.8 (11.6)	11.0**	Follow-up, Post > Baseline
**WMS-III-VR**										
Immediate recall	70.4 (10.1)	74.1 (14.6)	79.8 (14.4)	4.6*	Follow-up > Post, Baseline	63.7 (14.5)	70.5 (12.5)	73.3 (17.3)	3.5	-
Delayed recall	37.8 (17.1)	58.7 (22.7)	66.4 (23.5)	25.8***	Follow-up > Post > Baseline	44.3 (20.3)	57.0 (18.7)	60.7 (25.3)	4.0*	Follow-up > Baseline
Recognition	42.7 (2.9)	43.3 (3.1)	42.6 (3.8)	1.0	-	41.0 (3.0)	42.5 (2.2)	40.5 (3.4)	2.1	-

Note. HKLLT, Hong Kong List Learning Test; WMS-III-VR, Visuospatial Reproduction Subtest of the Wechsler Memory Scale III. A Greenhouse-Geisser correction was applied for dependent variables violating the assumption of sphericity. Standard deviations are in parentheses. *p < 0.05, **p < 0.01, ***p < 0.001.

Comparable results were obtained in the MI group. The results of repeated measures ANOVA showed that the main effects of time were significant for all HKLLT indices, including total learning, 10-min and 30-min delayed recall, and recognition, *F*s ≥ 7.0, *p*s ≤ 0.003, $\eta_{\text{p}}^{2}$ ≥ 0.29. Paired *t*-tests showed that all indices significantly improved from baseline to post- and follow-up assessments, *p*s < 0.02, *d*s ≥ 0.64. There were no significant differences between post- and follow-up, *p*s > 0.05. In addition, the main effect of time was significant for the WMS-III-VR immediate and delayed recall, *F*s ≥ 4.6, *p*s < 0.02, $\eta_{\text{p}}^{2}$ ≥ 0.21. Paired *t*-tests showed that visual immediate recall significantly improved from baseline to follow-up assessment, and from post- to follow-up assessment, *p*s ≤ 0.04, *d*s ≥ 0.53. Furthermore, visual delayed recall significantly improved from baseline to post- and follow-up assessments, *p*s ≤ 0.001, *d*s ≥ 1.4. No significant results were found for visual recognition, *p*s > 0.05.

**Figure 1:**
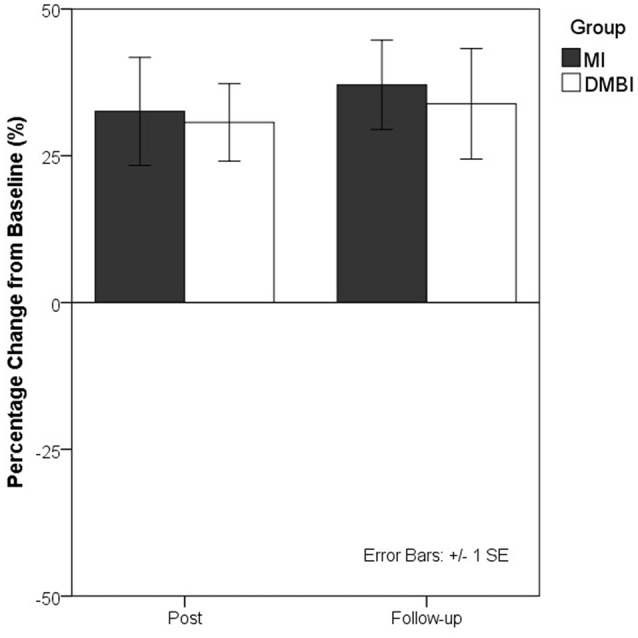
Percentage changes in the 10-min delayed recall on the Hong Kong List Learning Test (HKLLT) from baseline to post-intervention assessment (Post) and follow-up assessment (Follow-up) in the Dejian Mind-body Intervention (DMBI; *n* = 10) and Memory Intervention (MI; *n* = 16) groups. One participant from the DMBI group and two participants from the MI group were excluded because they were identified as extreme outliers by boxplots.

Because medium-to-large effect sizes were obtained for all HKLLT and WMS-III-VR measures that exhibited significant changes at the post- and follow-up assessments, as compared with baseline, the sustained memory-enhancing effects of both interventions appear to be substantial. Previous studies have persistently shown that short-term delayed recall on verbal list learning tests is one of the most reliable neuropsychological predictors of dementia (Tierney et al., 2005; Gainotti et al., 2014). To further illustrate the memory-enhancing effects of the DMBI and MI, Figure 1 shows the percentage of performance change from baseline for the 10-min delayed recall on the HKLLT in the DMBI and MI groups.

We found significant results for within-group but not between-group comparisons. To determine the minimum sample sizes required to achieve sufficient power for detecting group differences, we performed *post hoc* sample size calculations. These calculations were based on the effect sizes (i.e., Cohen’s *d*s) of group differences in sustained memory improvements (i.e., follow-up minus baseline), a statistical power of 0.80 and a significance level of 0.05. For all verbal memory measures (*d*s from 0.03 to 0.43), a sample size greater than 87 participants per group is needed to achieve sufficient power; for all visual memory measures (*d*s from 0.01 to 0.57), a sample size greater than 49 participants per group is needed.

Next, we evaluated the participants’ subjective impressions of changes in their current memory performance based on their ratings immediately and 18 months after the intervention. Results of the one-sample *t*-test showed that, at follow-up assessment, both the DMBI, *t*_(10)_ = 2.4, *p* = 0.038, and MI groups, *t*_(17)_ = 7.4, *p* < 0.001, reported significant improvement in their memory performance. In addition, an independent sample *t-test* showed no significant difference between the DMBI and MI groups at follow-up, *p* > 0.05. However, in the DMBI group, the subjective improvement at follow-up was less pronounced than that at post-assessment, *t*_(10)_ = 3.1, *p* = 0.01. There was no significant difference between the post- and follow-up assessments in the MI group, *p* > 0.05.

### Changes in Subjective Physical and Psychological Health

**Figure 2:**
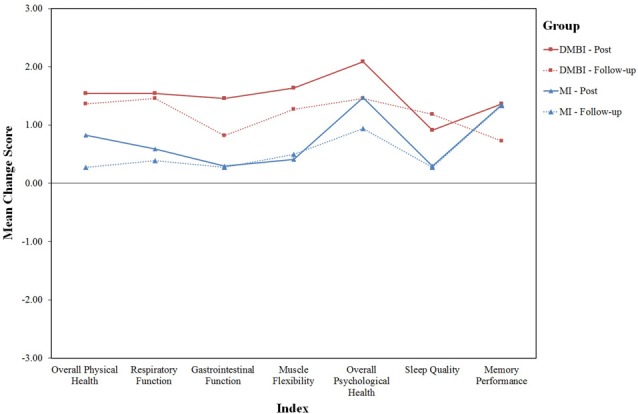
Mean score of six indices indicating changes in subjective physical and psychological health (i.e., Overall Physical Health, Respiratory Function, Gastrointestinal Function, Muscle Flexibility, Overall Psychological Health and Sleep Quality)in the DMBI (*n* = 11; red) and MI (*n* = 18; blue) groups at post-assessment (solid lines) and at follow-up assessment (dotted lines). One participant in the MI group was excluded at post-assessment because of missing data.

Finally, we examined how participants perceived the intervention they participated 18 months ago affected their current physical and psychological health (see Figure 2). One-sample *t*-test results showed significant improvements in all six indices for the DMBI group, including Overall Physical Health, *t*_(10)_ = 4.0, *p* = 0.002, Respiratory Function, *t*_(10)_ = 4.0, *p* = 0.003, Gastrointestinal Function, *t*_(10)_ = 2.3, *p* = 0.04, Muscle Flexibility, *t*_(10)_ = 3.3, *p* = 0.008, Overall Psychological Health, *t*_(10)_ = 4.0, *p* = 0.003, and Sleep Quality, *t*_(10)_ = 3.6, *p* = 0.005. In addition, the MI group showed significant improvements in only three of these indices, including Muscle Flexibility, *t*_(17)_ = 2.3, *p* = 0.04, Overall Psychological Health, *t*_(17)_ = 3.6, *p* = 0.002, and Sleep Quality, *t*_(17)_ = 2.6, *p* = 0.02. Importantly, the DMBI group had significantly higher scores than the MI group for Overall Physical Health, *t*_(14.4)_ = 2.9, *p* = 0.01, Respiratory Function, *t*_(27)_ = 2.7, *p* = 0.01 and Sleep Quality, *t*_(12.3)_ = 2.6, *p* = 0.02.

As indicated by the paired-sample *t-*tests used to compare participants’ subjective changes in physical and psychological health between the post- and follow-up assessments, the MI group reported a greater level of improvement in Overall Physical Health at post- compared to follow-up assessment, *t*_(16)_ = 2.9, *p* = 0.01. However, the DMBI group showed no significant differences in any of the six indices, *p*s > 0.05. Thus, there were generally no significant differences in self-rated changes between the post- and follow-up assessments in both groups.

### Daily Application of Intervention Methods at Follow-Up

At follow-up, we asked participants whether they continued to apply intervention methods in daily life. For the DMBI group, all of the participants self-reported currently adopting a lifestyle that incorporated at least one of the three major elements of the DMBI. Specifically, all of them were still regularly practicing *Nei Gong* (i.e., mind-body exercises) to facilitate *Qi* and blood circulation; 91% of them had modified their diets to reduce taking in food that generates excess internal heat; 46% of them were still practicing *Chan* to develop an understanding of the root of problems in accordance with the Buddhist philosophy. For the MI group, all but one participant reported continuing to apply mnemonic strategies in daily life. Specifically, 94% of them were still using external memory aids, such as reminder notes, and categorization. In addition, 44%, 56% and 39% of them were currently using internal mnemonic strategies such as spaced retrieval, association and visual imagery, respectively. In summary, almost all participants continued to apply intervention methods in daily life 18 months following completion of the intervention program.

Both the DMBI and MI involved teaching of multiple methods, and there were individual differences in the number of methods they were still practicing at follow-up. A recent meta-analysis showed that the number of methods (i.e., mnemonic strategies) taught or practiced was positively related to the effect size of memory improvement in older adults (Gross et al., 2012). Thus, to further clarify the potential basis underlying the sustained effects of the interventions, we performed analyses to determine whether continued practice of a larger number of methods (DMBI: range from 1 to 3; MI: range from 0 to 5) was associated with better memory functioning at follow-up. Memory functioning was indexed by the 10-min delayed recall on the HKLLT because this measure has been shown to be one of the most reliable neuropsychological predictors of dementia (Tierney et al., 2005; Gainotti et al., 2014; also see “Changes in Memory Functioning” section). After controlling for performance at baseline, partial correlation results showed that the number of methods still applied tended to positively correlate with the 10-min delayed recall on the HKLLT at follow-up for the DMBI group, *r*s_(8)_ = 0.57, *p* = 0.085, but not for the MI group, *p* = 0.28. Thus, the results suggest that older adults who self-reported applying the DMBI methods more fully tended to have better memory functioning at follow-up. Individual differences in the pattern of continued practice may play a role in the sustained effects of the DMBI.

## Discussion

The present study was the first longitudinal, randomized-controlled trial to assess and compare the effectiveness and durability of a comprehensive lifestyle intervention (i.e., the DMBI) with those of a conventional MI on the memory functioning of older adults with memory complaints. Specifically, we found that both intervention groups improved verbal immediate and delayed recall performance at follow-up compared with baseline. No significant differences in verbal memory performance between the post-intervention and follow-up periods were found, suggesting that verbal memory performance in both groups was enhanced and maintained across 18 months after the intervention. In addition, both intervention groups showed significant improvement in visual delayed recall at follow-up compared with baseline. Furthermore, participants in both groups perceived that the intervention they participated in 18 months ago brought improvements to their present memory performance and many aspects of their physical and psychological wellness. Importantly, the DMBI group reported greater physical health improvements compared to the MI group. Hence, the findings suggest an 18-month durability of both interventions on memory functioning and subjective physical and psychological wellness.

These results extend previous findings on the short-term benefits of the DMBI on improving memory functioning and self-perceived psychological and physical wellness in older adults (Chan et al., 2014, 2017; Yu et al., 2014). They also align with Metternich et al. (2008), who suggested that MI was the most effective intervention in enhancing objective memory of older adults with memory complaints. Additionally, the present study indicates the sustained benefits of the DMBI on older adults with memory complaints, whose gains were at least comparable to those who received a conventional MI. Thus, the memory-enhancing effects of the DMBI implicate its possibility as an alternative intervention for older adults to facilitate their memory functioning in the long-run. It is worth noting that most of the older adults in the present study received less than 12 years of education. Because the DMBI required less reading and writing efforts than the MI, the DMBI could be a more suitable intervention for older adults with limited levels of education.

The sustained effects of the DMBI and MI could be mediated by several mechanisms. First, almost all the older adults (i.e., DMBI: 100%; MI: 94%) self-reported continuing to apply intervention methods in daily life at the 18-month follow-up. Thus, the sustained effects of the DMBI and MI might be due to a continued practice of these methods over the 18-month post-intervention period. In addition, based on this continued practice, it is likely that the memory improvements measured at follow-up were still the effect of the intervention. Note that the memory improvements found at follow-up could be due to the after-effect of intervention, continued practice of the intervention method, or a combination of both. Because there was only one older adult who self-reported discontinued practice at follow-up, the present study could not compare the effects of intervention between those with and without continued practice. Nevertheless, the primary objective of the present study was to study the sustained effects of the 10-week DMBI and MI programs on memory in older adults. High rates of continued practice among older adults at follow-up suggest that the two interventions were well received by the older adults. Finally, based on principles of neural plasticity, the sustained effects of the DMBI and MI might be mediated by changes in the older adults’ brains that enhance reserve in the memory domain (Franzmeier et al., 2017a, b) as a result of environmental enrichment (i.e., intervention; Freret et al., 2015; Gelfo et al., 2018).

Mnemonic memory training, including the present MI, has been widely adopted for improving memory functioning of older adults (Belleville et al., 2006; Troyer et al., 2008; Jean et al., 2010; Vermeij et al., 2016). Thus, it is worthwhile to compare the present interventions’ effect sizes with those reported by previous studies. According to a meta-analytic study (Gross et al., 2012), the memory-enhancing effect of memory training interventions was of medium size (i.e., 0.43 *SD*). The mean retest effect in inactive control groups was of very small size (i.e., 0.06 *SD*). In the present study, the effect sizes of sustained improvements (i.e., follow-up minus baseline) for verbal memory measures ranged from 1.15–1.38 *SD* in the DMBI group and from 0.64 to 1.27 *SD* in the MI group. In addition, the effect sizes of sustained improvements for visual memory measures except for the WMS-III-VR recognition score ranged from 0.64 to 0.74 *SD* in the DMBI group and from 0.63 to 1.41 *SD* in the MI group. Thus, the effect sizes for almost all the memory measures ranged from medium to large in both the DMBI and MI groups. These effect sizes were above and beyond those expected for mnemonic memory training and those for inactive control.

The effects of the DMBI and MI might be large for several reasons. First, based on self-reported current practice of intervention methods at follow-up, it is likely that the older adults had been practicing the intervention methods over the 18-month post-intervention period. As a result of this continued practice, the intervention effect was maintained or even strengthened over time. In addition, the DMBI and MI involved multiple lifestyle changes and multiple mnemonic techniques, respectively. These interventions, which adopt a holistic approach, may lead to greater training gains than those focusing on isolated methods (Gross et al., 2012). Indeed, we found that older adults who applied the DMBI methods more fully tended to have better memory functioning at follow-up as compared to those who applied the methods less fully. This finding suggests that the number of intervention methods under continued practice might mediate the sustained effects of the DMBI.

Despite the observed improvements resulting from the DMBI and MI, the present study has several limitations. First, we could only recruit a proportion of older adults from the original DMBI and MI samples to participate in follow-up assessments. Even so, there was no significant difference in the number of participants who returned for a follow-up assessment between the DMBI and MI groups. There were neither demographic nor intervention-related differences between older adults who returned and those who did not. Thus, the current samples appear to be representative of the original samples. Second, the sample size was small. *Post hoc* sample size calculations showed that 87 and 49 individuals per group would be needed to achieve sufficient power for detecting group differences in sustained improvements in certain aspects of verbal and visual memory, respectively. Because these required sample sizes are within the range expected for practical significance (i.e., *n* < 91 per group; Ferguson, 2009), there is a possibility that the lack of group differences in sustained memory improvements was due to insufficient statistical power. Although it is encouraging to see the significant and medium-to-large effect sizes for the memory-enhancement retained in these samples, there is a clear need for future research to include a larger, and possibly more diverse sample to assess the overall sustained effects of the DMBI and to compare these effects with those of MIs in older adults.

In conclusion, the present study provides evidence that both the conventional MI and a Chinese lifestyle intervention appear to be effective in bringing both short-term and sustained benefits to memory functioning of older adults with memory complaints. Given that the world’s population is aging, both interventions may be options for reducing the risk for the development of dementia (Tierney et al., 2005; Gainotti et al., 2014) and relieving the pressure of the increased psychological distress, healthcare costs, and lower quality of life related to memory complaints (Comijs et al., 2002; Mol et al., 2008; Waldorff et al., 2009; Ito et al., 2013; Steinberg et al., 2013).

## Author Contributions

AC contributed to the conceptualization of the study. WC, MY and TLL contributed to the subject recruitment and data collection. All authors undertook the data analysis, prepared the initial draft of the manuscript and read and approved the final version of the manuscript.

## Conflict of Interest Statement

The authors declare that the research was conducted in the absence of any commercial or financial relationships that could be construed as a potential conflict of interest.
